# A novel mutation in the cathepsin C (CTSC) gene in Iranian family with Papillon‐Lefevre syndrome

**DOI:** 10.1002/cre2.387

**Published:** 2021-02-14

**Authors:** Mahmoud Ghanei, Mohammad R. Abbaszadegan, Mohammad M. Forghanifard, Azadeh Aarabi, Hamidreza Arab

**Affiliations:** ^1^ Medical Genetics and Molecular Medicine Department Medical school, Mashhad University of Medical Sciences Mashhad Iran; ^2^ Medical Genetics Research Center, Medical School Mashhad University of Medical Sciences Mashhad Iran; ^3^ Department of Biology, Damghan Branch Islamic Azad University Damghan Iran; ^4^ Human Genetics Division Immunology Research Center, Avicenna Research Institute, Mashhad University of Medical Sciences Mashhad Iran; ^5^ Dental Research Center, School of Dentistry Mashhad University of Medical Sciences Mashhad Iran

**Keywords:** cathepsin C mutation, energy minimization, Lefevre syndrome, palmoplantar hyperkeratosis, Papillon, tertiary structure

## Abstract

**Objectives:**

In this study, we analyzed the whole exomes of CTSC gene in a family with history of PLS.

**Materials and methods:**

Genomic DNA was extracted from peripheral blood and genotype analysis was performed. The mutated protein sequence was used to find the best possible tertiary structure for homology modeling. The homology modeling of the novel mutation was then performed using the online Swiss‐Prot server. The results were also analyzed for to verify its validity.

**Results:**

The analysis of CTSC gene elucidated a novel insertion GAC. The novel mutation was proved by analyzing 50 healthy control volunteers. Modeling of the novel found mutation in CTSC gene revealed structural defects that may have caused the functional abnormalities.

**Conclusions:**

The structural analysis of the mutated protein model identifies changes in the stereo‐chemical and the energy level of the mutated protein. Since this protein play a role in the activation of granule serine proteases from cytotoxic T lymphocytes, natural killer cells, mast cells, such structural defects may lead to its malfunction causing dysfunctioning of immune defense mechanisms.

## 1 INTRODUCTION

Papillon–Lefevre syndrome (PLS; OMIM245000) is a rare inherited autosomal recessive disorder firstly reported by French physicians Papillon and Lefevre in 1924. According to the latest reports, the prevalence of this syndrome is 1–4 in a million people with equal distribution in males and females (Cury et al., 2002; R J Gorlin, Sedano, & Anderson, 1964; Basapogu Sreeramulu, Shyam, Ajay, & Suman, 2015). Although, a variety of studies has recently reported different affected Iranian families with PLS, there is not any accurate incidence of PLS in Iranian families (Arnold, Bordoli, Kopp, & Schwede, 2006; Farjadian, Kiyanimanesh, Abbaszadegan, & Lotfazar, 2007; Farjadian, Lotfazar, & Ghaderi, 2008; Moghaddasian et al., 2014). The clinical symptoms of the syndrome is normally revealed in the first 4 years of patient life (It may be already present in the first 3 months of life however generally the palmoplantar hyperkeratosis and the severe periodontitis present simultaneously between 1 and 4 ages) (Farkas et al., 2013; Papillon, 1924; B Sreeramulu, Haragopal, Shalini, Sudha, & Kiran, 2012). This disorder is characterized by a symmetrical palmar‐plantar hyperkeratosis—dry scaly patches on the palms and the soles's skin—and severe inflammation and degeneration of the structures surrounding and supporting the teeth (periodontium) (Robert J Gorlin, 2000). Usually, complete deciduous tooth loss occurs before the 6 years of birth and then, the periodontitis disappears until all permanent teeth are fully grown out. All permanent teeth are normally lost when the patients turn 15 years old (Robert J Gorlin, Cohen Jr, & Hennekam, 2001; Wu et al., 2016).

Other symptoms of PLS are hyperhidrosis, intracranial calcification, arachnodactyly, increased susceptibility to infections, hearing loss, mental retardation, oculocutaneous albinism, bent nail syndrome, corneal cell hyperkeratosis, acanthosis nigricans, thicker cutaneous granular layer, etc (Almuneef, Al Khenaizan, Al Ajaji, & Al‐Anazi, 2003; Borroni et al., 1985; Farkas et al., 2013; Haneke, 1979; Khandpur & Reddy, 2001; Oğuzkurt, Tanyel, Büyükpamukçu, & Hiçsönmez, 1996; Basapogu Sreeramulu et al., 2015; Tosti, Manuzzi, Bardazzi, & Costa, 1988; Wani, Devkar, Patole, & Shouche, 2006; Wu et al., 2016). Furthermore, this disorder may also be accompanied with other symptoms such as the skin, liver, kidneys and brain abscesses (Gorlin et al., 1964; Kanthimathinathan et al., 2013; Mercy, Singh, Ghorpade, Das, & Upadhyay, 2013; Morgan, Hannon, & Lakhoo, 2011).

Susceptibility to bacterial infections (often in respiratory tract) is approximately increased in 20–25% of patients with recurrent pyogenic infections as a result of decreased neutrophil, lymphocyte or monocyte functions in PLS patients(6,12). The quality of patient's life can be improved by medications for hyperkeratosis and periodontal care. However, periodontal management of PLS is still a big challenge for treatment (Lundgren, Crossner, Twetman, & Ullbro, 1996; Lundgren & Renvert, 2004; Tekin, Yucelten, Beleggia, Sarig, & Sprecher, 2016).

The cathepsin C (CTSC) gene is mapped on chromosome 11q14.2 and encodes a cysteine‐lysosomal protease called cathepsin C. Mutations in this gene, are responsible for Papillon–Lefevre syndrome (Hart et al., 1999; Toomes et al., 1999). Now, 91 CTSC mutations have been reported in PLS patients (http://www.hgmd.cf.ac.uk/ac/gene.php?gene=CTSC, n.d.; Pallos et al., 2010). Approximately more than half of these mutations are missense; however, a broad spectrum of mutation types have been described (Tekin et al., 2016).

Recently it has been revealed that Loss of function in lysosomal protease cathepsin C is the main genetic cause of the disease. This tetrameric protein plays a critical role in the removal of dipeptides from free N‐terminal of some hormones (including gastrin, glucagon and angiotensin II) and the activation of granule serine proteases from cytotoxic T lymphocytes, natural killer cells (granzymes A and B), mast cells (tryptase and chymase) and neutrophils (cathepsin G and elastase) (de Haar et al., 2004; Idon, Olasoji, & Fusami, 2015; Romero‐Quintana et al., 2013; Toomes et al., 1999).

Since 1999, several mutations have been reported in the CTSC gene majorly related to PLS. Herein, we present the case of a 6‐year‐old boy affected by insertion GAC (p.122ins.T) in the third exon of the CTSC gene.

## 2 MATERIAL AND METHODS

### 2.1 Patients

This study was performed on a PLS patient (a 6 years old boy) and his parents. The symptoms of the patient became apparent at the age of four. Periodontium and palmar plantar hyperkeratosis were observed in the patient and his parents. Loss of deciduous teeth was observed at the same time as the onset of symptoms in the patient, however, loss of permanent teeth was not seen until the age of six. Susceptibility to infections was another symptom seen in the patient. The declared consent form was obtained from all members of the family. The study was approved by the Ethics Committee of Mashhad University of Medical Sciences (# 910523).

### 2.2 Genotype analysis

Genomic DNA was extracted from peripheral blood leukocytes of the proband as well as his parents. Genotype analysis was performed on extracted DNA using specific primer sets for all seven exons of CTSC gene (Moghaddasian et al., 2014). Both strands of PCR products were directly sequenced using ABI Prism 310 Automated Sequencer and analyzed with the DNA sequence assembly software (Sequencher 4.10.1, Gene Codes Corporation). Numbering of CTSC nucleotides and amino acids was in accordance with the reference sequence on Gene Bank (NM_001814). Fifty healthy volunteers were also analyzed as a control group.

### 2.3 Structural analysis and protein modeling

To model the newly identified mutation (p.122insT), the mutated CTSC amino acid sequence was compared to template databases using the online server Swiss‐Prot Template Identification tool (Arnold et al., 2006; Biasini et al., 2014; Kiefer, Arnold, Künzli, Bordoli, & Schwede, 2008) on http://swissmodel.expasy.org.

The most similar template to the target mutated protein, with 100% sequence identity, was human cathepsin C, which has been solved to 2.15 Å 1K3BA including the amino acids of the first chain of the protein (Kiefer et al., 2008). Other structures with more sequence coverage in terms of the number of the amino acids than 1K3BA structure were also found, but the X‐ray structure of the 1K3BA was selected for two reasons; Firstly, the complete sequence identity with the mutated region, and secondly, its better quality for the purpose of the homology modeling that can exactly describe the structural defects caused by the mutation.

The selected 1K3BA structure was submitted for homology modeling using the online Swiss‐Prot server for automated modeling on http://swissmodel.expasy.org (Biasini et al., 2014; Guex & Peitsch, 1997; Schwede, Kopp, Guex, & Peitsch, 2003). The result was set for the Energy Minimization job using ZMM software. The ZMM uses the Amber all‐atom force field with a cut‐off distance of 10 Å and Monte Carlo Minimization Method to minimize conformational energy in the space of generalized coordinates including torsions and bond angles (Zhenqin Li & Scheraga, 1987; Zdobnov & Apweiler, 2001). The Energy Minimization was terminated after 100 sequential minimizations failed to improve the lowest‐energy conformation.

The essential accuracy and correctness of the model was then evaluated using the PROCHECK and WHAT‐IF programs from the online server at http://nihserver.mbi.ucla.edu/SAVES/ (Vriend, 1990).

The electrostatic potential of the molecule was computed using Coulomb's Law and the Swiss‐PdbViewer 4.02, as well as the graphical representations presented here (Guex & Peitsch, 1997).

## 3 RESULTS

**FIGURE 1:**
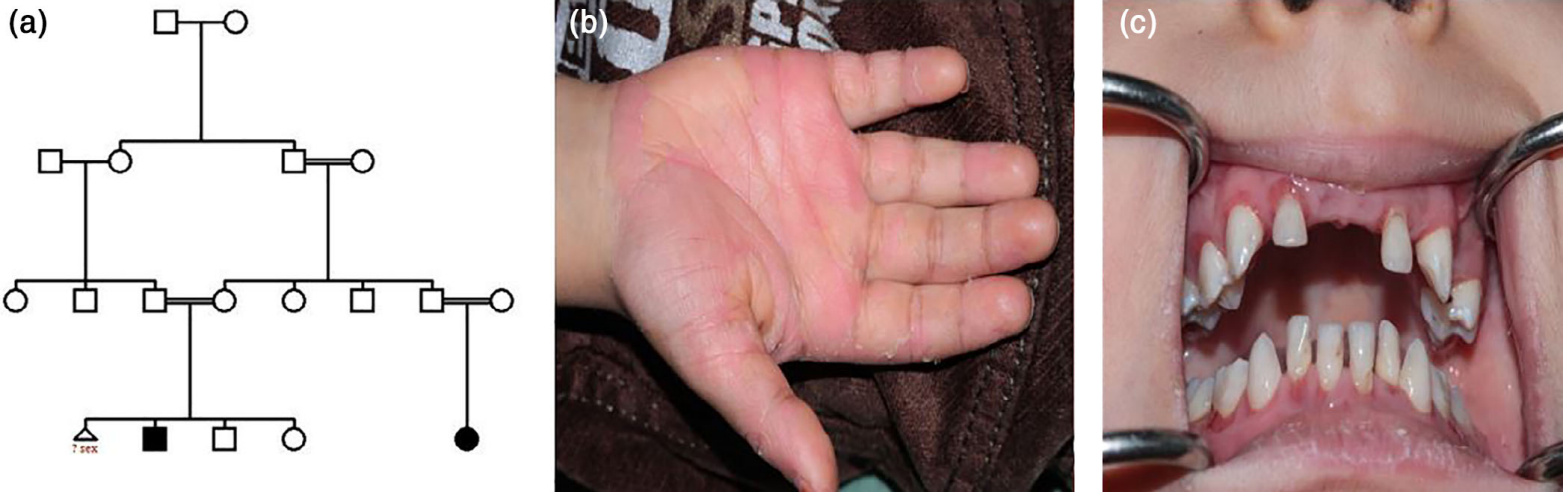
A 6‐year‐old boy as only affected one in his family; he has an affected cousin too. Pedigree (a) and clinical manifestation (b, c) have been shown here

**FIGURE 2:**
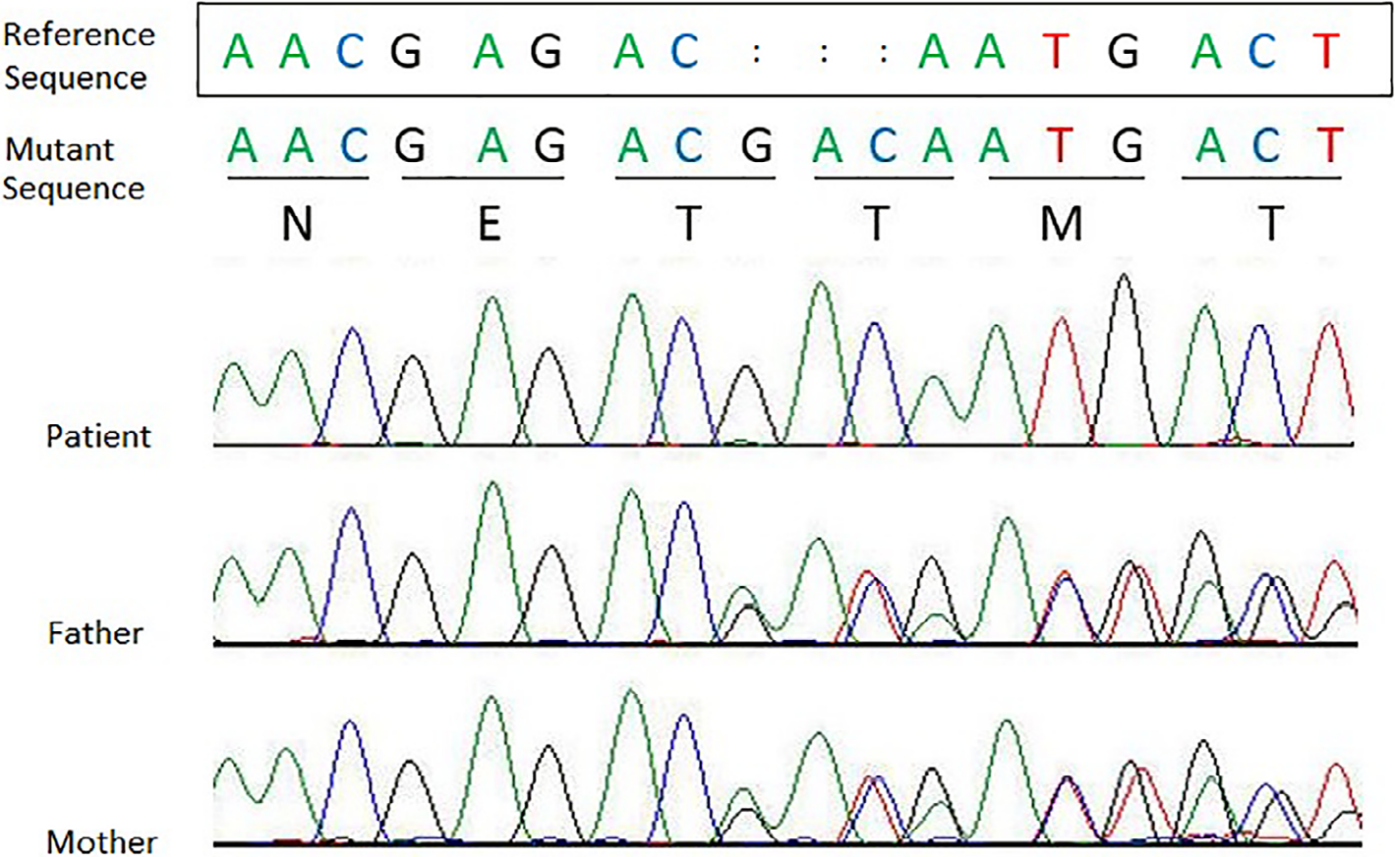
Identification of a novel mutation of the CTSC gene. Direct automated sequencing revealed a three‐nucleotide insertion (GAC) in the exon 3 of the CTSC gene. Affected person carried the mutation in homozygous form while the unaffected father and mother showed heterozygous form

A 6‐year‐old boy that was diagnosed as Papillon–Lefevre syndrome affected (according to the established criteria by Gorlin et al.) was referred to Avicenna Research Institute with chief complain of a long history of hyperkeratosis and periodontitis. The majority of his deciduous teeth fell off by the age of 6 years. The family history revealed that he was born from a relative parents and the only affected one of the family (Guex & Peitsch, 1997; Zhiming Li et al., 2014). His cousin shows clinical symptoms of the disease but his siblings seem normal at the time of study (Figure 1a, b and c). The complete analysis of coding sequences and splice sites of CTSC gene revealed a novel three nucleotides GAC insertion (p.122insT) in the third exon of the gene in‐patient in homozygous form, which lead to the insertion of an additional threonine in polypeptide chain. Further genotype analyses of parents showed the existence of mentioned mutation in heterozygous form (Figure 2). All the healthy control volunteers showed the wild type sequence of the gene.

**FIGURE 3:**
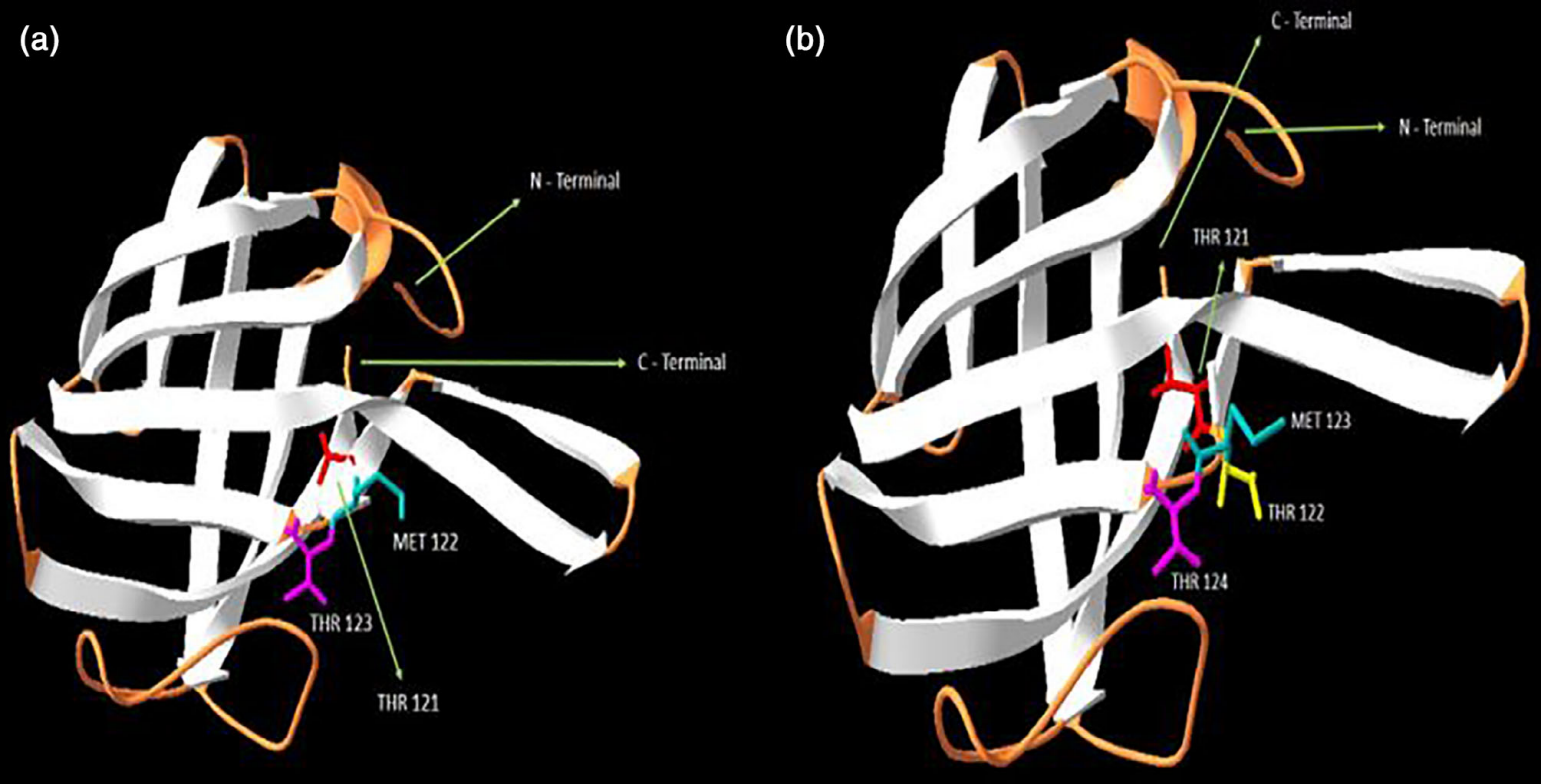
The normal whole‐view of the three‐dimensional structure of the 1K3BA, showing the exclusion domain of Dipeptidyl peptidase I (cathepsin C) (a) and the mutated whole‐view of the three‐dimensional structure of the 1K3BA, showing the mutated exclusion domain of Dipeptidyl peptidase I (cathepsin C) (b)

The tertiary structures of the normal and mutated proteins are shown in Figure 3. The model was also analyzed in terms of stereo‐chemical and geometrical parameters such as G‐Factor, bond length, and bond angles, for which the results be satisfied with the discussed criteria. In addition, most of the residues were inside the favorable regions of the Ramachandran map. After energy minimization, the overall energy of the model was −666.19 kcal/mol for the 1K3BA structure.

Structural changes that have been emerged by the insertion of the Threonine amino acid in the normal gene (p.122ins.T), as predicted from molecular modeling, are compatible with the observed phenotype.

## 4 DISCUSSION

Although PLS is a rare disease, its psychological and social impacts on growing affected children can influence the quality of their life. The exact etiology of PLS is still ambiguous. However, Anatomy, microorganisms, immunologic response and genetic factors are responsible for development of the syndrome. Some microbial agents including Actinobacillus actinomycetemcomitans was reported as a significant factor in development of periodontal involvements (Stabholz, Taichman, & Soskolne, 1995). Recent investigations have demonstrated that PLS is mostly seen in consanguineous marriages, which is directly related to the cathepsin C abnormality. Dipeptidyl peptidase I (DPPI) or cathepsin C has a vital role in defense against pathogenic organisms by physiological activating of some serine proteases in immune cells. Therefore, deficiency of cathepsin C function will be resulted in lack of immunological response, leading to increased risk of severe infections (Basapogu Sreeramulu et al., 2015). The CTSC gene that code cathepsin C, is commonly expressed in epithelial cells and causes different clinical symptoms such as severe gingivostomatitis and periodontitis. Early diagnosis of PLS has important role in management of patient's oral conditions.

In this study, we report a novel mutation in CTSC gene, which can lead to production of an abnormal protein according to the obtained results of DNA genomic sequencing and in Silico studies.

Genetic testing of studied family revealed presence of the novel insertion in homozygous form in the third exon of CTSC gene (p.122ins.T) of affected patient. Up till now 85% of PLS patients are in homozygous form in CTSC mutations which 13% are located in exon 1 to 3, located in the exclusion domain of the related protein (Nagy et al., 2014). Coding sequence of cathepsin c contains 463 amino acids including a signal peptide with 24 amino acids, an exclusion domain with 233 amino acids and a propeptide with 206 amino acids that it also contains the heavy and light chain regions (Nitta et al., 2005). The C‐terminal portion of the exclusion domain is a conserved region, which is thought to be important for enzyme activity. Mutation in this region blocks access to the active site and inhibits enzyme to bind substrates.

Structural analyses of the mutated protein also revealed important changes in its tertiary structure due to the elucidated three nucleotides insertion (p.122ins.T) in the active site of Dipeptidyl peptidase I domain (cathepsin C), exclusion domain, SSF75001, accessed by InterproScan (Zdobnov & Apweiler, 2001). As in Figure 3a, which is related to the normal protein model, the amino acids threonine, methionine, and threonine are at positions 121, 122, and 123 in exclusion domain of the protein, respectively, but after the mutation (p.122ins.T), a threonine after threonine 121 is added. Proximity of three threonines can also alter the protein structure, leading to poor or inappropriate protein function; this change is shown in Figure 3b. Therefore, due to the strong relationships between the protein tertiary structure and its functions, it can be realized that such important abnormality in the structure of the mutated protein can be cause consequent important abnormalities in the protein function.

In conclusion, according to the results obtained from this study, we can justify correlation between this reported mutation and observed phenotype in this 6‐year‐old‐boy affected by Papillon–Lefevre syndrome.

## CONFLICT OF INTEREST

The authors have stated explicitly that there are no conflicts of interest in connection with this article.

## CLINICAL SIGNIFICANCE

Scientific rational for study: To identify the genes and mutations causing the disease, the patient's genome and its parents were sequenced. Analysis of the sequencing results showed homozygous mutation in the CTSC gene in the patient and heterozygote in his parents, which was then confirmed by sanger sequencing which these results were in compliance with PLS.

Principal findings: We identified a novel mutation (p.122insT) in CTSC gene that can cause Papillon–Lefevre syndrome. Computational analysis of this variant confirm it as a novel mutation.

Practical implications: By conducting further functional studies and confirming this variant as mutation, clinicians and geneticists that analyze the CTSC gene sequences, can consider this variant as a mutation and provides appropriate recommendations for their patients.

## Data Availability

The data that support the findings of this study are available from the corresponding author upon reasonable request.

## References

[cre2387-bib-0001] Almuneef, M., Al Khenaizan, S., Al Ajaji, S., & Al‐Anazi, A. (2003). Pyogenic liver abscess and Papillon‐Lefèvre syndrome: Not a rare association. Pediatrics, 111(1), e85–e88. 12509601 10.1542/peds.111.1.e85

[cre2387-bib-0002] Arnold, K., Bordoli, L., Kopp, J., & Schwede, T. (2006). The SWISS‐MODEL workspace: A web‐based environment for protein structure homology modelling. Bioinformatics, 22(2), 195–201. 16301204 10.1093/bioinformatics/bti770

[cre2387-bib-0003] Biasini, M., Bienert, S., Waterhouse, A., Arnold, K., Studer, G., Schmidt, T., … Bordoli, L. (2014). SWISS‐MODEL: Modelling protein tertiary and quaternary structure using evolutionary information. Nucleic Acids Research, 42(W1), W252–W258. 24782522 10.1093/nar/gku340 PMC4086089

[cre2387-bib-0004] Borroni, G., Pagani, A., Carcaterra, A., Pericoli, R., Gabba, P., & Marconi, M. (1985). Immunological alterations in a case of Papillon‐Lefèvre syndrome with recurrent cutaneous infections. Dermatology, 170(1), 27–30. 10.1159/000249491 3156059

[cre2387-bib-0005] Cury, V. F., Costa, J. E., Gomez, R. S., Boson, W. L., Loures, C. G., & De Marco, L. (2002). A novel mutation of the cathepsin C gene in Papillon‐Lefevre syndrome. Journal of Periodontology, 73(3), 307–312. 10.1902/jop.2002.73.3.307 29539009

[cre2387-bib-0006] de Haar, S. F., Jansen, D. C., Schoenmaker, T., De Vree, H., Everts, V., & Beertsen, W. (2004). Loss‐of‐function mutations in cathepsin C in two families with Papillon‐Lefèvre syndrome are associated with deficiency of serine proteinases in PMNs. Human Mutation, 23(5), 524. 10.1002/humu.9243 15108292

[cre2387-bib-0007] Farjadian, S., Kiyanimanesh, N., Abbaszadegan, A., & Lotfazar, M. (2007). HLA class I gene polymorphism in Iranian patients with Papillon‐Lefevre syndrome. Iranian Journal of Immunology, 4(4), 241–245. 18057583 10.22034/iji.2007.17204

[cre2387-bib-0008] Farjadian, S., Lotfazar, M., & Ghaderi, A. (2008). Analysis of human leukocyte antigen class II gene polymorphism in Iranian patients with Papillon‐Lefevre syndrome: A family study. Iranian Journal of Immunology, 5(3), 171–176. 18791284 10.22034/iji.2008.17163

[cre2387-bib-0009] Farkas, K., Paschali, E., Papp, F., Vályi, P., Széll, M., Kemény, L., … Csoma, Z. (2013). A novel seven‐base deletion of the CTSC gene identified in a Hungarian family with Papillon‐Lefèvre syndrome. Archives of Dermatological Research, 305(5), 453–455. 23397598 10.1007/s00403-013-1323-z

[cre2387-bib-0010] Gorlin, R. J., Sedano, H., & Anderson, V. E. (1964). The syndrome of palmar‐plantar hyperkeratosis and premature periodontal destruction of the teeth: A clinical and genetic analysis of the Papillon‐Lefèvre syndrome. The Journal of Pediatrics, 65(6), 895–908. 14244097 10.1016/s0022-3476(64)80014-7

[cre2387-bib-0011] Gorlin, R. J. (2000). Of palms, soles, and gums. Journal of Medical Genetics, 37(2), 81–82. 10662805 10.1136/jmg.37.2.81 PMC1734520

[cre2387-bib-0012] Gorlin, R. J., Cohen, M. M., Jr., & Hennekam, R. C. M. (2001). Syndromes of the head and neck. Oxford, England: Oxford University Press.

[cre2387-bib-0013] Guex, N., & Peitsch, M. C. (1997). SWISS‐MODEL and the Swiss‐Pdb viewer: An environment for comparative protein modeling. Electrophoresis, 18(15), 2714–2723. 9504803 10.1002/elps.1150181505

[cre2387-bib-0014] Haneke, E. (1979). The Papillon‐Lefèvre syndrome: Keratosis palmoplantaris with periodontopathy. Human Genetics, 51(1), 1–35. 159254 10.1007/BF00278288

[cre2387-bib-0015] Hart, T. C., Hart, P. S., Bowden, D. W., Michalec, M. D., Callison, S. A., Walker, S. J., … Firatli, E. (1999). Mutations of the cathepsin C gene are responsible for Papillon‐Lefevre syndrome. Journal of Medical Genetics, 36(12), 881–887. 10593994 PMC1734286

[cre2387-bib-0016] http://www.hgmd.cf.ac.uk/ac/gene.php?gene=CTSC . (n.d.). Retrieved from http://www.hgmd.cf.ac.uk/ac/gene.php?gene=CTSC.

[cre2387-bib-0017] Idon, P., Olasoji, H., & Fusami, M. (2015). Papillon‐Lefèvre syndrome: Review of literature and report of three cases in the same family. The Nigerian Postgraduate Medical Journal, 22(1), 75–82. 25875417

[cre2387-bib-0018] Kanthimathinathan, H. K., Browne, F., Ramirez, R., McKaig, S., Debelle, G., Martin, J., … Moss, C. (2013). Multiple cerebral abscesses in Papillon–Lefèvre syndrome. Child's Nervous System, 29(8), 1227–1229. 10.1007/s00381-013-2152-2 23686359

[cre2387-bib-0019] Khandpur, S., & Reddy, B. S. N. (2001). Papillon–Lefèvre syndrome with pyogenic hepatic abscess: A rare association. Pediatric Dermatology, 18(1), 45–47. 11207971 10.1046/j.1525-1470.2001.018001045.x

[cre2387-bib-0020] Kiefer, F., Arnold, K., Künzli, M., Bordoli, L., & Schwede, T. (2008). The SWISS‐MODEL repository and associated resources. Nucleic Acids Research, 37(suppl_1), D387–D392. 18931379 10.1093/nar/gkn750 PMC2686475

[cre2387-bib-0021] Li, Z., & Scheraga, H. A. (1987). Monte Carlo‐minimization approach to the multiple‐minima problem in protein folding. Proceedings of the National Academy of Sciences, 84(19), 6611–6615. 10.1073/pnas.84.19.6611 PMC299132 3477791

[cre2387-bib-0022] Li, Z., Liu, J., Fang, S., Zhu, H., Zhang, X., Cai, J., … Xu, Y. (2014). Novel compound heterozygous mutations in CTSC gene cause Papillon–Lefèvre syndrome with high serum immunoglobulin E. Journal of Dermatological Science, 76(3), 258–260. 25450092 10.1016/j.jdermsci.2014.09.009

[cre2387-bib-0023] Lundgren, T., Crossner, C., Twetman, S., & Ullbro, C. (1996). Systemic retinoid medication and periodontal health in patients with Papillon‐Lefèvre syndrome. Journal of Clinical Periodontology, 23(3), 176–179. 8707975 10.1111/j.1600-051x.1996.tb02073.x

[cre2387-bib-0024] Lundgren, T., & Renvert, S. (2004). Periodontal treatment of patients with Papillon–Lefèvre syndrome: A 3‐year follow‐up. Journal of Clinical Periodontology, 31(11), 933–938. 15491306 10.1111/j.1600-051X.2004.00591.x

[cre2387-bib-0025] Mercy, P., Singh, A., Ghorpade, A. K., Das, M., & Upadhyay, A. (2013). Papillon‐Lefèvre syndrome: Two siblings, one developing liver abscess. Indian Journal of Dermatology, 58(5), 410. 10.4103/0019-5154.117361 PMC3778820 24082225

[cre2387-bib-0026] Moghaddasian, M., Arab, H., Dadkhah, E., Boostani, H., Babak, A. R., & Abbaszadegan, M. R. (2014). Protein modeling of cathepsin C mutations found in Papillon–Lefèvre syndrome. Gene, 538(1), 182–187. 24374475 10.1016/j.gene.2013.11.079

[cre2387-bib-0027] Morgan, R. D., Hannon, E., & Lakhoo, K. (2011). Renal abscess in Papillion–Lefèvre syndrome. Pediatric Surgery International, 27(12), 1381–1383. 21594717 10.1007/s00383-011-2931-3

[cre2387-bib-0028] Nagy, N., Vályi, P., Csoma, Z., Sulák, A., Tripolszki, K., Farkas, K., … Fábos, B. (2014). CTSC and Papillon–Lefèvre syndrome: Detection of recurrent mutations in H ungarian patients, a review of published variants and database update. Molecular Genetics & Genomic Medicine, 2(3), 217–228. 24936511 10.1002/mgg3.61 PMC4049362

[cre2387-bib-0029] Nitta, H., Wara‐aswapati, N., Lertsirivorakul, J., Nakamura, T., Yamamoto, M., Izumi, Y., … Ishikawa, I. (2005). A novel mutation of the Cathepsin C gene in a Thai family with Papillon‐Lefèvre syndrome. Journal of Periodontology, 76(3), 492–496. 15857086 10.1902/jop.2005.76.3.492

[cre2387-bib-0030] Oğuzkurt, P., Tanyel, F. C., Büyükpamukçu, N., & Hiçsönmez, A. (1996). Increased risk of pyogenic liver abscess in children with Papillon‐Lefevre syndrome. Journal of Pediatric Surgery, 31(7), 955–956. 8811566 10.1016/s0022-3468(96)90420-0

[cre2387-bib-0031] Pallos, D., Acevedo, A. C., Mestrinho, H. D., Cordeiro, I., Hart, T. C., & Hart, P. S. (2010). Novel cathepsin C mutation in a Brazilian family with Papillon‐Lefèvre syndrome: Case report and mutation update. Journal of Dentistry for Children, 77(1), 36–41. 20359428 PMC4617240

[cre2387-bib-0032] Papillon, M. (1924). Deux cas de keratodernune palmaire symmetrique famiale (Maladie de Meleda) chez Le frere et la soeur. Coexistece dans les deux cas d'alterations dentaires graves. Bulletin de la Société Française de Dermatologie et de Syphiligraphie, 31, 82–84.

[cre2387-bib-0033] Romero‐Quintana, J. G., Frías‐Castro, L. O., Arámbula‐Meraz, E., Aguilar‐Medina, M., Dueñas‐Arias, J. E., Melchor‐Soto, J. D., … Ramos‐Payán, R. (2013). Identification of novel mutation in cathepsin C gene causing Papillon‐Lefèvre syndrome in Mexican patients. BMC Medical Genetics, 14(1), 7. 23311634 10.1186/1471-2350-14-7 PMC3563609

[cre2387-bib-0034] Schwede, T., Kopp, J., Guex, N., & Peitsch, M. C. (2003). SWISS‐MODEL: An automated protein homology‐modeling server. Nucleic Acids Research, 31(13), 3381–3385. 12824332 10.1093/nar/gkg520 PMC168927

[cre2387-bib-0035] Sreeramulu, B., Haragopal, S., Shalini, K., Sudha, M. D., & Kiran, G. (2012). The prosthodontic management of a young edentulous patient with the papillon lefevre syndrome‐a rare case report. Journal of Clinical and Diagnostic Research: JCDR, 6(10), 1808–1811. 23373060 10.7860/JCDR/2012/4884.2607 PMC3552236

[cre2387-bib-0036] Sreeramulu, B., Shyam, N. D. V. N., Ajay, P., & Suman, P. (2015). Papillon–Lefèvre syndrome: clinical presentation and management options. Clinical, Cosmetic and Investigational Dentistry, 7, 75. 10.2147/CCIDE.S76080 PMC4507741 26203280

[cre2387-bib-0037] Stabholz, A., Taichman, N. S., & Soskolne, W. A. (1995). Occurrence of Actinobacillus actinomycetemcomitans and anti‐leukotoxin antibodies in some members of an extended family affected by Papillon‐Lefèvre syndrome. Journal of Periodontology, 66(7), 653–657. 7562358 10.1902/jop.1995.66.7.653

[cre2387-bib-0038] Tekin, B., Yucelten, D., Beleggia, F., Sarig, O., & Sprecher, E. (2016). Papillon–Lefèvre syndrome: Report of six patients and identification of a novel mutation. International Journal of Dermatology, 55(8), 898–902. 27062382 10.1111/ijd.13297

[cre2387-bib-0039] Toomes, C., James, J., Wood, A. J., Wu, C. L., McCormick, D., Lench, N., … Woods, C. G. (1999). Loss‐of‐function mutations in the cathepsin C gene result in periodontal disease and palmoplantar keratosis. Nature Genetics, 23(4), 421–424. 10581027 10.1038/70525

[cre2387-bib-0040] Tosti, A., Manuzzi, P., Bardazzi, F., & Costa, A. (1988). Is etretinate dangerous in Papillon‐Lefèvre syndrome? Dermatology, 176(3), 148–150. 10.1159/000248692 2967778

[cre2387-bib-0041] Vriend, G. (1990). WHAT IF: A molecular modeling and drug design program. Journal of Molecular Graphics, 8(1), 52–56. 2268628 10.1016/0263-7855(90)80070-v

[cre2387-bib-0042] Wani, A. A., Devkar, N., Patole, M. S., & Shouche, Y. S. (2006). Description of two new cathepsin C gene mutations in patients with Papillon‐Lefevre syndrome. Journal of Periodontology, 77(2), 233–237. 16460249 10.1902/jop.2006.050124

[cre2387-bib-0043] Wu, W., Chen, B., Chen, X., Chen, L., Yi, L., Wang, Y., … Sun, W. (2016). A novel large deletion combined with a nonsense mutation in a C hinese child with P apillon–L efèvre syndrome. Journal of Periodontal Research, 51(3), 376–380. 26385525 10.1111/jre.12317

[cre2387-bib-0044] Zdobnov, E. M., & Apweiler, R. (2001). InterProScan–an integration platform for the signature‐recognition methods in InterPro. Bioinformatics, 17(9), 847–848. 11590104 10.1093/bioinformatics/17.9.847

